# On Anasarca in Disease of the Heart

**Published:** 1851-10

**Authors:** 


### PATHOLOGY AND PRACTICE OF MEDICINE.

*On Anasarca in Disease of the Heart.* By M. CHOMEL.—The progress of infiltration is ordinarily slow and progressive in affections of the heart; but, nevertheless, nothing is more common than to meet with individuals among the working-classes, who, while presenting the appearance of health, and without having manifested any sign of disease, are seized with anasarca, the physical and material signs of cardiac alterations not being present, or only, at all events, to a very slight degree. This is because there are causes prevailing in this class of society,—such as excess of labor, fatigue, watchings, misery, drinking,—which, in a measure, precipitate the course of the disease. These causes come in addition to the natural influence of the disease; and the anasarca appears at a period when without these it would not have manifested itself. So, when these causes are removed, and the patient is kept at rest, and sheltered from the unfortunate conditions that have given rise to so serious a complication, the œdema diminishes daily, and the patient soon leaves the hospital believing himself cured. New exposure to excesses, fatigue, or misery, reproduce the anasarca, which may be again dispersed, and that for several times; but after a certain number of such attacks, it in the end becomes permanent.

Frequently the appearance of an acute anasarca, throws a ray of light on obscure and embarrassing cases, indicating in a great majority of cases an acute disease of the heart. Doubtful endocarditis and pericarditis are often thus revealed to the observer by general œdema. M. Chomel thus considers that in the case of anasarca coming on, when we can discover neither change in the blood nor albumen in the urine, we are authorized in admitting the existence of disease of the heart or large vessels, even when all material signs of this affection are completely absent.—*British and Foreign Review, from L'Union Médicale,* 1851, No. 26.

